# Could Antigen Presenting Cells Represent a Protective Element during SARS-CoV-2 Infection in Children?

**DOI:** 10.3390/pathogens10040476

**Published:** 2021-04-14

**Authors:** Rita Lauro, Natasha Irrera, Ali H. Eid, Alessandra Bitto

**Affiliations:** 1 Department of Clinical and Experimental Medicine, University of Messina, 98125 Messina, Italy; rita.lauro@studenti.unime.it (R.L.); nirrera@unime.it (N.I.); 2 Department of Basic Medical Sciences, College of Medicine, QU Health, Qatar University, P.O. Box 2713, Doha, Qatar; 3 Biomedical and Pharmaceutical Research Unit, QU Health, Qatar University, P.O. Box 2713, Doha, Qatar

**Keywords:** Antigen Presenting Cells (APC), SARS-CoV-2, COVID-19, juvenile immunity, cytokine storm, IFN-signaling

## Abstract

Antigen Presenting Cells (APC) are immune cells that recognize, process, and present antigens to lymphocytes. APCs are among the earliest immune responders against an antigen. Thus, in patients with COVID-19, a disease caused by the newly reported SARS-CoV-2 virus, the role of APCs becomes increasingly important. In this paper, we dissect the role of these cells in the fight against SARS-CoV-2. Interestingly, this virus appears to cause a higher mortality among adults than children. This may suggest that the immune system, particularly APCs, of children may be different from that of adults, which may then explain differences in immune responses between these two populations, evident as different pathological outcome. However, the underlying molecular mechanisms that differentiate juvenile from other APCs are not well understood. Whether juvenile APCs are one reason why children are less susceptible to SARS-CoV-2 requires much attention. The goal of this review is to examine the role of APCs, both in adults and children. The molecular mechanisms governing APCs, especially against SARS-CoV-2, may explain the differential immune responsiveness in the two populations.

## 1. Introduction

In December 2019, several cases of pneumonia with unknown etiology were identified in Wuhan, China. These cases of pneumonia were later associated with a novel virus called Severe Acute Respiratory Syndrome Coronavirus-2 (SARS-CoV-2). On 11 March 2020, the World Health Organization announced that SARS-CoV-2 was causing the disease Coronavirus Disease (COVID-19), now considered a pandemic. This disease is characterized by respiratory tract infections that, depending on the severity, can precipitate acute respiratory stress disorder. Since this pathological outcome can lead to lung injury and multi-organ dysfunction, elders with medical problems have a higher risk of developing the worst symptomatology, while children have been sporadically affected [1].

SARS-CoV-2 is a single-stranded-RNA virus that belongs to the family of coronaviruses, characterized by the peculiar presence of spike-like projections on their surface that gives them a crown-like appearance [2]. Coronaviruses usually circulate among animals, but can sometimes “jump” to other species, including humans. This process is called “spillover event”, and seems that it happened in Wuhan with SARS-CoV-2 [3].

SARS-CoV-2 bears many similarities to other coronaviruses like Severe Acute Respiratory Syndrome Coronavirus (SARS-CoV) and Middle East Respiratory Virus Syndrome Coronavirus (MERS-CoV), which respectively share 79% and 50% sequence identity to SARS-CoV-2. Notably, SARS-CoV causes Severe Acute Respiratory Syndrome (SARS), and MERS-CoV causes Middle East Respiratory Syndrome (MERS) [4]. Both syndromes are respiratory diseases that have been considered as epidemics. Because of the resemblance between the different coronaviruses, identifying their differences and similarities is instrumental for a better understanding of the novel SARS-CoV-2.

The principal products encoded by SARS-CoV-2 genome are two large poly-proteins, named pp1a and pp1ab, and four groups of structural proteins: S (spike), E (envelope), M (membrane and accessory proteins), and N (nucleocapsid) proteins [5]. All these proteins are important for the establishment of the virus-host interactome as well as for the virus survival.

**Figure 1:**
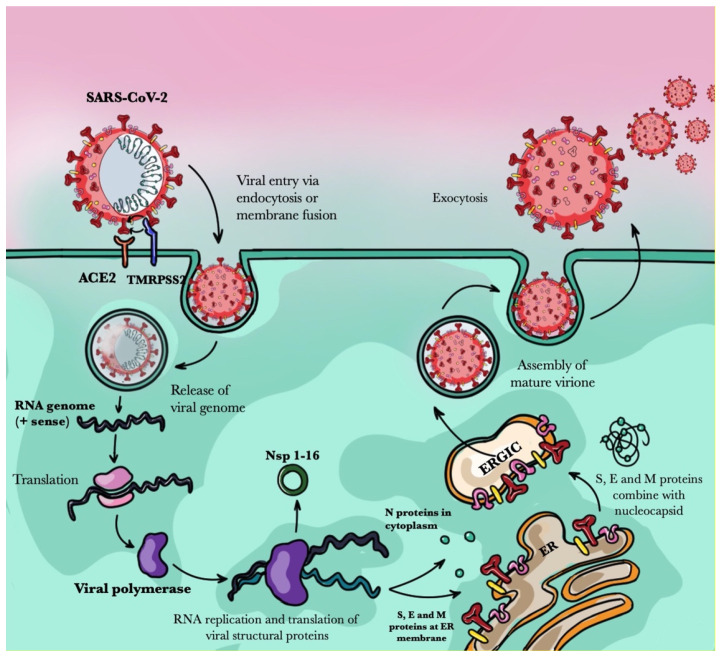
Host cell entry mechanism of SARS-CoV-2. Angiotensin-converting Enzyme 2 (ACE2) and Transmembrane Protease/Serine subfamily 2 (TMRPSS2) are proteins in the host cell surface that are exploited by SARS-CoV-2 viral spike (S) protein for its entry. Once inside the host cell, the viral genome of SARS-CoV-2 is released, uncoated, and translated to form the viral replication and transcription complex. The SARS-CoV-2 structural proteins—namely, spike (S), envelope (E), and membrane (M)—are translocated to the Endoplasmic Reticulum (ER) and then to the Endoplasmic Reticulum-to-Golgi Intermediate Compartment (ERGIC), while the nucleocapsid (N) protein is released into the cytoplasm once translated and interacts with the newly produced genomic viral RNA. Passing through the ERGIC, S, E, and M proteins combine with the nucleocapsid, assembling a mature virion that is secreted from the cell via exocytosis.

Pp1a and pp1ab are involved in viral replication. They are processed into 16 non-structural proteins, named nsp1-nsp16, which form the viral complex of the replicase transcriptase. The other proteins, S, E, M, and N, are implicated in viral infection and replication [5]. The S protein is vital for SARS-CoV-2 entry into host cells because it recognizes and binds to the cell surface protein Angiotensin-Converting Enzyme 2 (ACE2) [3]. The S protein mediates viral attachment to the host cell and membrane fusion through its subunits, S1 and S2. S1, which encompasses the Receptor Binding Domain (RBD), specifically recognizes the peptidase domain (PD) of ACE2, whereas S2 is important for the membrane fusion. After S1 binds to the host ACE2, proteolytical cleavage is needed in order to fuse membranes [6]. Indeed, TMPRSS2, a host serine-protease, cleaves a site at the S1/S2 boundary, inducing a conformational change in the S2. This cleavage leads to viral infection via endocytosis or direct fusion of the viral envelope with the host membrane [7] (Figure 1).

The virulence ability of SARS-CoV-2 is achieved by the E proteins, which are small hydrophobic membrane proteins that can form cation-selective ion channels [8,9]. Indeed, when new virions are produced, E proteins promote Ca^2+^ transport activity at the ER-Golgi Intermediate Compartment (ERGIC). This increase in Ca^2+^ concentration in the ERGIC could activate NLRP3 inflammasome [10], which is a multiprotein complex that induces an inflammatory form of cell death and the release of pro-inflammatory cytokines, as IL-1β. This latter is critical for the host response to infections, and can trigger an auto-inflammatory loop that promotes the typical COVID-19 hyper-inflammatory status [11,12].

M proteins are viral envelope and accessory proteins. They interact with N proteins, which tightly bind to the viral RNA genome, packing it into a nucleocapsid [13]. This interaction enhances genome condensation, nucleocapsid stabilization, and viral envelope shape. In addition to being the most abundant proteins in coronaviruses, N proteins are also highly immunogenic and are implicated in the modulation of several cell signaling pathways. This suggests that one of SARS-CoV-2 immune escape mechanisms could be accomplished by the N protein, contributing to the disease’s pathogenesis [14].

SARS-CoV-2 surface epitopes are recognized by the host immune system. A recent study highlighted a T helper response against S, M, and N proteins in samples from 20 convalescing COVID-19 patients [15]. In COVID-19, the humoral immune response is primarily directed against the S-RBD region [16] and the N proteins [17]. Moreover, even in SARS-CoV-2-infected pediatric patients, neutralizing antibodies directed against the same proteins were also observed, but in a lower number than adults. [18,19]. Interestingly, recognition of these antigenic structures and the proper activation of T lymphocytes by APCs is necessary. In the below section, we discuss the role of APCs in the immune response against SARS-CoV-2.

## 2. Innate Immunity Response to SARS-CoV-2

Entry of SARS-CoV-2 into host cells activates the immune response via pattern recognition receptors (PRRs) of APCs. These PPRs recognize pathogen-associated molecular patterns (PAMPs), which are molecular structures belonging to and produced by pathogens, as well as damage-associated molecular patterns (DAMPs), endogenous molecules produced or released by damaged cells. In SARS-CoV-2, viral RNA and spike proteins are the virus’s principal PAMPs [20]. It therefore becomes of particular interest to determine which PRRs respond to a viral infection like SARS-CoV-2’s.

In general, PRRs are divided into two major classes: membrane-bound and cytoplasmic. Viral infections can be detected by both membrane-bound PRRs, such as the family of Toll-Like Receptors (TLRs) or C-type Lectin-Like Receptors (CLRs), as well as by cytosolic ones, such as RIG-I-Like Receptors (RLRs) or NOD-Like Receptors (NLRs) [19]. When bound to an antigen, each PRR is able to induce a different response. The effectiveness of the molecular signaling is important for the proper activation of the immune system.

Interaction of a TLR with an antigen induces two TLRs to come into direct physical contact. This enhances the recruitment of different cytoplasmic molecular adapters, such as MyD88, known for promoting the activation of inflammatory pathways [21,22].

This recruitment initiates a signaling cascade that induces nuclear re-localization of cytoplasmic transcription factors such as NF-κB, IRF3, and IRF7, which are among the most critical factors responsible for COVID-19 progression [23]. In particular, NF-κB is a complex system of proteins in the cytoplasm which, once activated upon induction, can translocate to the nucleus and promote the expression of genes involved in both innate and adaptive immune response. NF-κB also plays a pivotal role in the establishment of the so-called cytokine storm, via MyD-88-dependent and independent pathways [24]. IRF3 and IRF7, on the other hand, are master regulators of the Type 1 IFN (IFN-1) response, a cellular line of defense which promotes viral clearance. They can also be activated upon phosphorylation induced by MyD88 or other molecular adapters of PRRs [25].

These factors promote expression programs related to antiviral responses, including signaling molecules involved in inflammatory response like cytokines, chemokines, adhesion molecules, and type I Interferons (IFN-1). These molecules, which are released from cells as a defensive response to a virus, are actually inhibited by SARS-CoV-2 [26].

TLRs have varying affinities to viral components. TLR3, for example, recognizes viral RNA and is highly expressed on dendritic cells (DCs). By activating NF-κB and IRF3 signaling pathways, this TLR enhances protective responses against SARS-CoV and MERS [27]. Similarly, TLR3, TLR7, and TLR8 recognize viral RNA and appear to be implicated in coronaviruses’ response. Once activated, these TLRs promote the MyD88 pathway, which increases the expression of inflammatory cytokines, especially IL-6, IL-12, TNF-α, and IFN-α. Interestingly, levels of these cytokines is usually very high in severe COVID-19 patients [28]. TLR4, on the other hand, recognizes oxidized phospholipids produced after SARS-CoV-2 infection and the ensuing hyper-inflammatory response.

C-type Lectin-Like Receptors (CLRs), which can bind the glycosylated moiety of S proteins, are also possibly involved in the response against SARS-CoV-2. Interestingly, these receptors are mostly expressed by APCs of air mucosa and lung tissue [29], suggestive of being a route to expedite SARS-CoV-2 spreading. This is especially so, since the interaction with such receptors promotes virus transfer to ACE2-expressing cells. Another important role of CLRs is modulation of TLR signaling as well as PRR-mediated responses [30].

Cytosolic receptors like RLRs can also recognize viral RNA [31]. For instance, one of these RLRs, namely RIG-1, binds to viral 5′-PPP RNA and short dsRNA, enhancing the function of NF-κB, IRF3, and IRF7. Although SARS-CoV-2 is a positive single-stranded RNA, it is likely to have similar replication intermediates which can be recognized by RLRs [32]. The signaling pathways emanating from these receptors lead to the production of inflammatory cytokines and IFN-1. While IFN-1 establishes an antiviral state through the expression of Interferon-Stimulated Genes (ISGs) [33], inflammatory cytokines promote systemic inflammation. In spite of the importance of the cytokines in promoting the immune system to eradicate a pathogen, imbalances in their production and/or activation of their receptors culminate in damaging inflammatory events [34]. Moreover, even if IFN-1 have an important role protecting against the infection, SARS-CoV-2 can inhibit its signaling by virtue of its ability to express multiple interferon antagonists [35].

### 2.1. Cytokines

Cytokines are a group of small signaling molecules secreted by immune cells [36]. They can act in autocrine, paracrine, or endocrine fashions. Moreover, they modulate the activity of other cytokines in an additive, synergistic, or antagonistic manner. Recent studies described the role of cytokines on host immune response during the SARS-CoV-2 infection. Some of these cytokines includes interferons (IFNs), interleukins (ILs), chemokines and Tumor Necrosis Factor (TNF) [36,37]. Some of these cytokines that are involved in SARS-CoV-2 are provided in the Table 1.

IFNs are a family of cytokines which play a primary role in defense against pathogens, particularly viruses. IFNs may be divided into three major classes: type 1, type 2 (IFN-2), and type 3 (IFN-3). Type 1, represented by IFNα and β, and type 3, represented by IFN-λ1 and IFN-λ2/3 subtype, are produced by innate immune cells after sensing pathogens components. They exhibit important antiviral effects primarily through the inhibition of viral replication. IFN-1 and IFN-3 also promote and amplify antigen presentation to T cells through the induction of MHC I expression by infected cells. On the other hand, IFN-II (represented only by IFN-γ) are secreted by activated lymphocytes and NK cells and have different immunomodulant properties both in innate and adaptive immune responses. Indeed, type 2 IFNs activate macrophages by upregulating MHC II molecules as well as genes involved in the phagocytosis. In this way, IFN-2 promotes direct antiviral and antimicrobial mechanism, as well as antigen presentation. Moreover, it regulates Immunoglobins (Igs) class switching in B lymphocytes to improve pathogens clearance [38,39,40].

ILs comprise a large family of cytokines, and are mainly involved in cell proliferation, maturation, differentiation, and may exert both pro- and anti-inflammatory effects [41]. Pro-inflammatory cytokines, such as IL-1β and IL-6, enhance the host defense against antigens through the promotion of inflammation, signaling the immune system to fight off invaders. Anti-inflammatory cytokines, such as IL-1Ra or IL-10, dampen the inflammatory cytokine response [42,43]. As a result of their ability to balance the inflammatory response, ILs may also modulate immune responses.

Chemokines are cytokines that promote chemotaxis or recruitment of responsive cells by guiding their migration. For instance, chemokines are needed for the migration of lymphocytes to the lymph nodes to interact with APCs, or for the recruitment of leukocytes to the site of infection [44].

TNFs comprise a superfamily of cytokines, of which TNF-α is the principal member, eliciting an important role in inflammation as well as in the equilibrium between survival and death of specific target cells [45]. Indeed, through the stimulation of a class of receptors containing death domains, TNF-α is responsible for promoting programmed cell death. TNF-α binds to other classes of receptors, allowing the transcription of adhesion molecules on vascular endothelial cells and inducing chemokine-mediated recruitment of leukocytes to the site of inflammation [46].

### 2.2. Type I IFN Response and SARS-CoV-2

Once the PRRs of APCs have recognized the viral structures, an antiviral, IFN-1 based program is established. IFNs produced at the early stage of SARS-CoV-2 infection can either bind to receptors present on the same cell and therefore protect this cell from subsequent infections, or they can bind to receptors on adjacent cells, inducing an antiviral state [4]. ISGs can then act at different levels, inhibiting the transcription of the viral RNA, its translation, or post-translational modifications, as well as its entry to other cells [47]. They can also induce pro-apoptotic genes, repress anti-apoptotic genes, and stimulate the expression of MHC I molecules to potentiate recognition of infected cells by the immune system. As such, expression of viral genes can be attenuated, and the virus replication inhibited [48].

IFN-1 and IFN-3 possess antiviral activity and share many immune activities [49]. However, their action and expression are more compartmentalized and restricted to certain cells, such as DCs, neutrophils, hepatocytes and tissue epithelial cells, typically those of the respiratory and gastrointestinal tracts [50]. IFN-3 binds to specific cell receptors, called Interferon Lambda Receptors (IFNLRs) [51], but the signaling pathways following their stimulation overlaps those triggered by IFN-1. Indeed, both IFN types can induce the transcription of the same overall repertoire of ISGs [52]. Nonetheless, type 3 IFNs are secreted at lower concentrations, thus providing a lower potency in the antiviral function. For this reason, type III IFNs are considered less potent than type I IFNs. However, ISGs expression by IFN-3 is more sustained and long-lasting, probably because of the intrinsic characteristics of the IFNLR signaling pathway [53,54]. Moreover, they induce less inflammatory damage, hence keeping the integrity of epithelial barriers. For this reason, their action can be more appreciated in infections with low viral loads. SARS-CoV-2, as well as other human coronaviruses, being highly pathogenic, induce a robust IFN-1 response to improve antiviral defenses [55,56]. Indeed, it has been demonstrated that viral load has to7 be considered the cardinal point determining the different contribution of the different types of IFNs [57].

In order to promoting their own survival and infection, several viruses including SARS-CoV-2 can, however, overcome IFN-mediated mechanisms [58]. Indeed, coronaviruses produce multiple interferon antagonists, such as the structural and nonstructural proteins like nsp1, nsp3, nsp13, nsp14, nsp15, nsp16, ORF3b, ORF6, M and N that interact with downstream signaling molecules of PRRs, resulting in a non-productive inflammation [59]. For example, nsp1, ORF6, and M protein inhibit the activity of different transcription factors related to the establishment of an inflammatory response [60]. Thus, during the early stage of infection, by overcoming IFN responses, SARS-CoV-2 promotes its dissemination and the continuous stimulus of an hyper-inflammatory innate immune response [61]. This is responsible for the pathophysiology of COVID-19, whose main symptom is a high titer of pro-inflammatory cytokines. Moreover, a recent study demonstrated that ACE2 is in fact a human ISG. This suggests that SARS-CoV-2 could exploit ACE2 upregulation interfering with IFN responses to promote infection [47].

As such, during the incubation phase, SARS-CoV-2 increases its viral load and impairs IFN response, eventually leading to inflammation and hypercytokinemia [62,63]. This is mainly due to the direct activation of NF-κB by the viral N protein, which then leads to increased release of pro-inflammatory cytokines and chemokines. More importantly, delayed IFN response enhances the recruitment of myeloid immune cells, such as monocyte-derived macrophages, leading to their accumulation and activation in lungs, aggravating cytokine secretion and impairing the pro-repair functions of airway macrophages [62]. Since IFN signaling also controls the activity of NK cells and stimulates T cells proliferation [64,65], its deficiency will precipitate other consequences in the early clearance of infected cells and in the establishment of an adaptive immune response. Taken together, the decreased antiviral response and hyper-inflammatory immune response can be considered the main causes for COVID-19 severity [66].

### 2.3. Cytokine Storm in SARS-CoV-2

COVID-19 disease is mostly characterized by the release of a great amount of inflammatory cytokines, an event known as cytokine storm. While pro-inflammatory cytokines promote pathogen clearance [67], their unbalanced release, along with an unregulated activation of their respective receptors, can precipitate a deleterious hyper-inflammation [68]. This hyper-inflammation is largely due to the activation of cellular PRRs and the consequent potentiation of NF-κB, IRF3, and IRF7.

**Figure 2:**
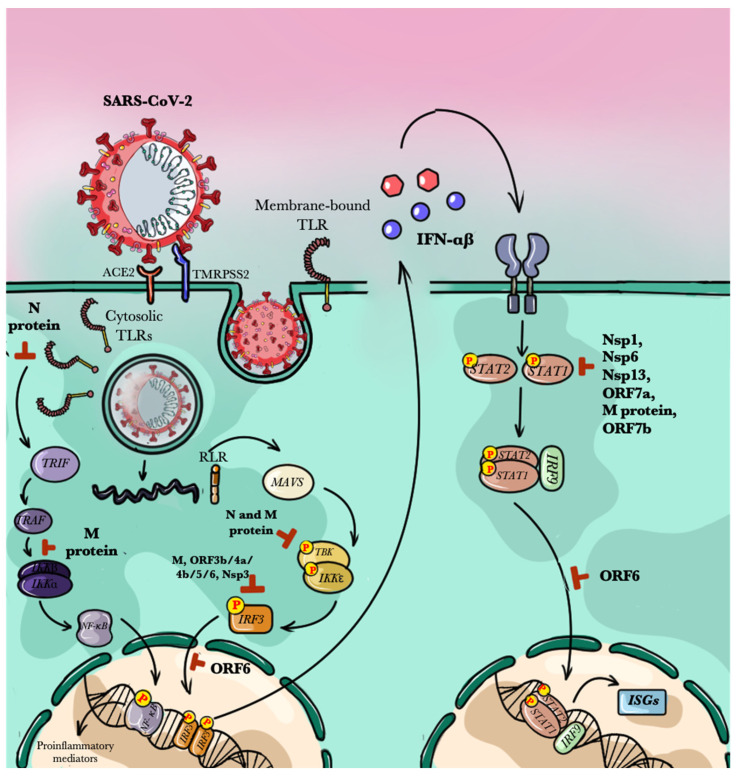
SARS-CoV-2 and host interactions and viral evasion strategies. Pattern Recognition Receptors (PRRs), localized in the host cell membrane and cytoplasm, sense the viral RNA, triggering the Interferon (IFN) and NF-κB pathways that lead to the expression of type I IFN (IFN-α and IFN-β) and pro-inflammatory mediators, like the pro-inflammatory cytokines. However, several components of SARS-CoV-2 can interfere with these mechanisms: N and M proteins impairs the TLRs activation through the inhibition of the recruitment of TRIF to the TLR and the inhibition of the nuclear translocation of NF-κB. They also interfere with the IFN-signaling, along with several viral ORFs and Nsp3. This leads to the reduced expression of Interferon Stimulated Genes (ISGs). Nsp1, Nsp6, Nsp13, ORF7a, M proteins, ORF7b and ORF6 can also inhibit the IFN signaling interfering respectively with the activity of the transcription factor STAT1 and its nuclear translocation.

In COVID-19 patients, IL-1, TNF-α, and IL-6 play critical roles in recruiting macrophages, neutrophils, and lymphocytes to the site of infection. This is then followed by a wave of pro-inflammatory cytokines that damage endothelial cells, the vascular barrier, and the alveoli [69]. In affected patients, the outcome of such mounted levels of cytokines is the development of Acute Respiratory Distress Syndrome (ARDS), a severe lung condition which can cause respiratory failure, a major cause of COVID-19 mortality [70]. The intense production of inflammatory cytokines, along with the early delayed production of IFN-1 due to SARS-CoV-2 immunoevasion mechanisms, may modulate some intrinsic functions of APCs. In particular, modulating their principal activity can compromise a virus-specific adaptive immunity response [71] (Figure 2).

## 3. Role of APCs in SARS-CoV-2

As a consequence of impaired adaptive immunity response, one of the most typical clinical features displayed by COVID-19 patients at the early onset of the disease is lymphopenia [72,73]. This is probably due to mechanisms of immune evasion mediated by SARS-CoV-2 [66].

It is known that the beginning of an adaptive response by T and B lymphocytes is elicited only if a proper antigen presentation by APCs is carried out. The ability to detect a pathogen almost instantly at its entry makes these cells the critical determinants of the viral pathogenicity. Interestingly, DCs are abundant in the respiratory tract, and they probably play a key role in the antigen presentation of SARS-CoV-2. However, SARS-CoV-2 infection is characterized by a diminished activity of DCs, both in acute and chronic COVID-19 cases [74], and infected DCs do not exhibit an IFN response, probably due to the viral inhibition of the transcription factor STAT1 [75,76]. As such, the early capacity to eradicate the pathogen is severely limited.

Decreased production of IFN-1 by DCs undermines their ability to limit the initial viral replication and as a consequence weakens the activity of NK cells. Because NKs destroy virus-infected cells [77], responses of innate immunity are severely suppressed when NK activities are attenuated. Moreover, low production of IFN-1 evokes reduced maturation of other DCs. This reduction leads to an unsuccessful T cell activation, and a failure in initiating a proper response against the virus. Moreover, altered levels of cytokines and chemokines due to an altered response in DCs’ activity induce a massive up-regulation of chemotactic messengers, as well as cytokine and chemokine receptors [78]. This event may drive a high recruitment of inflammatory cells, resulting in hyper-inflammation. Indeed, despite the lack of an IFN response, pro-inflammatory molecules seem to be widely expressed by DCs, especially the IP-10 chemokine, whose increased levels are associated with the exacerbation of ARDS [79].

In addition to DCs, macrophages are key producers of pro-inflammatory cytokine, and, accordingly, autopsies of COVID-19 patients revealed a more consistent presence of macrophages and monocytes [80]. As a matter of fact, SARS-CoV-2 can infect monocyte and macrophages in both an ACE2 dependent and independent way. The latter is possible due to the viral binding on cell surface molecules, such as CD147, DC-specific intercellular adhesion molecule 3-grabbing-non-integrin (DC-SIGN)lor Liver/Lymph node-SIGN (L-SIGN) [81].

In monocytes and macrophages infected by SARS-CoV-2, IFN-mediated responses are suppressed because of the inactivation of the transcription factor IRF-3. This leads to an alteration in the secretion of key pro-inflammatory molecules [14]. Impaired IFN-responses could also be due to the interaction of SARS-CoV-2 protein Nsp5 with an epigenetic regulator that regulates MHC II expression, which then limits antigen presentation and cytokine production [82,83,84]. Indeed, an excessive upregulation of neutrophils and monocyte-attractant chemokines was reported in COVID-19 patients [85]. These chemokines play a crucial role in promoting local inflammation and contribute to the clinical outcome of SARS-CoV-2 infection [80], potentiating the cytokine storm.

In macrophages, SARS-CoV-2 could also induce a form of cell death called pyroptosis via a viral accessory protein known as ORF-8 [86]. Indeed, this protein largely determines the activation of NLRPs in SARS-CoV infections and leads to the secretion of IL-1β, an inductor of pyroptosis [87]. Given that ORF-8 is highly similar in both SARS-CoV and SARS-CoV-2, it is possible that similar mechanisms occur in macrophages of COVID-19 patients [26]. However, this requires more research to be validated.

**Figure 3:**
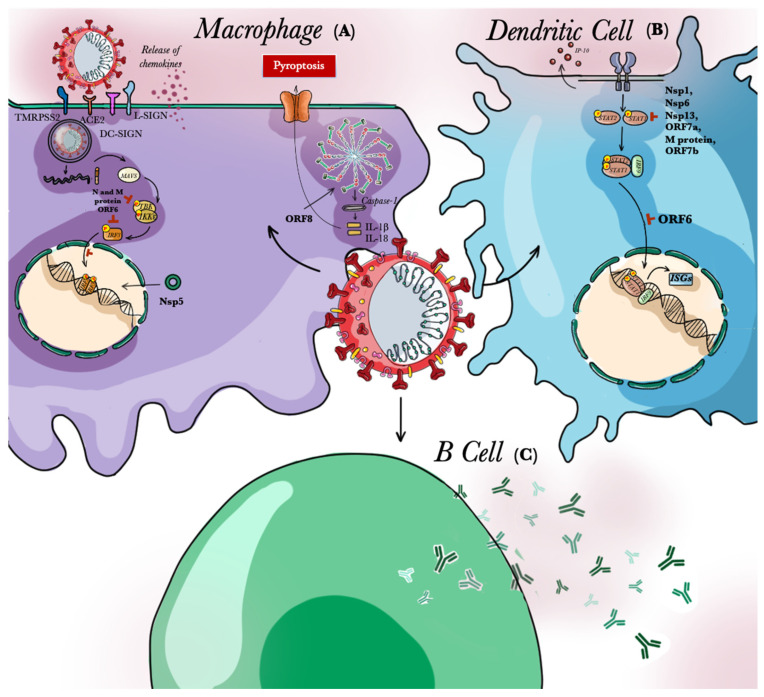
Schematic representation of the activity of Antigen Presenting Cells (APCs) infected with SARS-CoV-2. A macrophage (**A**), a Dendritic Cell (**B**) and a B cell (**C**) are represented, as well as the effects that SARS-CoV-2 induces in their activity. (**A**) In Macrophages, the entry of SARS-CoV-2 can also be mediated DC-specific intercellular adhesion molecule 3-grabbing-non-integrin (DC-SIGN) or Liver/Lymph node-specific intercellular adhesion molecule 3-grabbing integrin (L-SIGN). The viral ORF6 inactivates the transcription factor IRF3, suppressing IFN-mediated responses and inducing an altered secretion of pro-inflammatory mediators. This results in an excessive release of chemokines, which attracts pro-inflammatory cells and other monocytes, also promoting other cells infection. Nsp5, instead, acts against an epigenetic regulator which regulates the expression of Major Histocompatibility Complexes II (MHC II), limiting antigen presentation. Moreover, the ORF8 induces pyroptosis through the activation of the inflammasome. (**B**) In Dendritic Cells, the inhibition of the activity of STAT1 reduces the expression of ISGs and increases the expression of the chemokine IP-10. This leads to the recruitment of inflammatory cells, and also alters their capacity to activate T cells. (**C**) B cells release proteins against N and S proteins.

In COVID-19 patients, B cells release virus-specific antibodies that last for at least 12 weeks, and this is followed by release of virus-specific IgGs that lasts for longer times [88,89]. This is one way by which B cells are decisive components in the resolution of the disease [89]. Higher titers of antibodies against SARS-CoV-2 N- and S-proteins are reported in severely affected patients. Indeed, severe COVID-19 patients have been successfully treated with convalescent plasma, which appears to improve lung function and reduce the inflammatory response [90]. Now that the number of convalescent patients has increased, the FDA issued on 23 August 2020 an emergency use authorization for the use of this approach to treat hospitalized patients. Nevertheless, after the isolation and characterization of blood samples from eight different COVID-19 patients, a surprising new report identified anti-SARS-CoV-2 neutralizing antibodies which could be considered species-specific viral inhibitors. The crystal structure analysis revealed that their binding to the SARS-CoV-2 RBD causes a steric hindrance which inhibits viral particles from binding to ACE2 receptors. These findings suggest that these neutralizing antibodies may be new candidates for the development of new treatments for SARS-CoV-2 [91] Figure 3.

## 4. APCs and Children Response to SARS-CoV-2: Any Relation?

Molecular mechanisms governing APCs’ activity following SARS-CoV-2 infection, and suggests a possible incapacity of these cells to inhibit the spreading of the virus. This consequently plays a central role in the establishment of the most common features of COVID-19. Interestingly, although people of all ages are susceptible to SARS-CoV-2, previously healthy children seem to sustain a milder form of COVID-19, resulting in lower morbidity or mortality.

However, different hypotheses were suggested to explain the lower susceptibility of children. It was proposed that, compared to adults, children have a lower density or different conformation of ACE2 in their airways [92,93,94]. Therefore, these differences could then mean that the probability of its binding are reduced, leading to a lower spreading of the virus. Interestingly, ACE2 expression increases with age, with adults showing higher expression than adolescents, who in turn has haveh igher expression than children [93].

Another hypothesis suggests that the immature immune system of children and could be helpful in developing a less violent specific immune response. Ultimately, an important role of cross-protection was shownb y vaccinations was hypothesized. For example, and it was shown that BCG vaccination, a vaccine primarily used against tuberculosis, could be responsible for an increased expression of PRRs [95]. This could be beneficial in facilitating the detection and elimination of the virus or even allowing a certain amount of antiviral response despite viral immuno-evasion mechanisms [96]. This is due to the fact that innate immune cells may have a memory phenotype following the encounter with an antigen, a phenomenon referred to as trained immunity (TRIM). Contextually, metabolic, mitochondrial and epigenetic reprogramming prime immune cells to better respond to different pathogens for as long as they last [97]. Thus, frequent vaccination of children could decrease the risk of SARS-CoV-2 infections in children.

Despite the fact that SARS-CoV-2 infection has a mild or uncomplicated course in juveniles, multisystem inflammatory syndrome in children (MIS-C) is a rare clinical manifestation which may arise in the pediatric COVID-19 patients [98,99,100]. MIS-C usually occurs four to six weeks after the infection, and it is marked by an increase in inflammatory markers and consequent high fever as well as organ dysfunction, partially suggestive of Kawasaki disease. MIS-C is not characterized by severe respiratory symptoms, and it has a clinical outcome that is very different from ARDS, which could be developed by adults following SARS-CoV-2 infection. The different innate immune response could be responsible for the development of such different conditions, following the same infection, in adults and children [101]. Another glancing difference between adults and children infected by SARS-CoV-2 is represented by the reduced antibody response in children. As a matter of fact, adults produce high titers of anti-spike (S) IgG, IgM, and IgA, and anti-nucleocapsid (N), whereas children commonly display a lower level of specific antibodies [15,102,103]. Children predominantly generate anti-S IgG, with lower titers of anti-N IgG than the adults, low titers of anti-S IgM and varying titers of anti-S IgA antibodies.

It should be pointed out that the lack of nucleocapsid-specific antibodies suggests a lower spreading of the virus in juveniles [19]. This evidence implies a more robust innate immune response in children, which does not need the boost of a strong adaptive response [104]. This consideration is also supported by the fact that a strong T cell response for SARS-CoV-2 is not usually developed in children.

For all the above-mentioned reasons, the different nature of the innate immune response should be considered a determinant of the organism response to the virus. Particularly, the primitive status of children’s immune system should be considered as a potential significant contributor to their lower susceptibility to SARS-CoV-2. Indeed, this system is often considered too immature to properly fight most diseases, but immunological immaturity, and consequently APCs’ immaturity, could be considered a benefit in those diseases characterized by excessively uncontrolled immune response. For instance, neonatal APCs show immature markers, and often inhibited by regulatory T cells, and produce low levels of cytokines. Since the main symptom of COVID-19 is the establishment of an uncontrolled cytokine storm, it could be hypothesized that the presence of immature APCs in juveniles may be responsible for a mild inflammatory response that is less severe compared to adults. In fact, children are not immune to the virus; they just control it better. Interestingly, neonatal DCs are characterized by lower MHC II levels and co-stimulatory molecules with the consequent reduction of endocytic activity, which could decrease the number of viral particles involved in the entry, infection, and presentation to T cells [105]. Neonatal DCs show a defective production of cytokines in response to TLR activation, probably because of some differences in its downstream signaling pathway [106]. Contextually, T cell co-stimulation after antigen presentation, needed for APC full activation, is weak, with a consequent reduced clinical severity of SARS-CoV-2 infection in children. However, these diminished activities are usually overcome with age [106,107].

In this context, SARS-CoV-2 infected juvenile DCs expressed slightly higher levels of IFN genes than adult DCs. This could promote a more efficient antiviral response in children even during COVID-19, assisting the viral clearance of infected cells and reducing the number of cell infiltrate in lungs [78]. Juvenile macrophages can also play an important role in children immune response against SARS-CoV-2. They are characterized by a defective downstream signaling pathways of TLRs, where a lower expression of MyD88 decreases the production of TNF-α and IL-6 [108].This could be due to the role of macrophages at the earliest stages of life, when they are mainly necessary for tissue remodeling rather than immune response [109]. Moreover, neonatal monocytes are also capable of acting like suppressor cells towards T lymphocytes, since they have a distinct reactivity to inflammatory stimuli as compared to adult monocytes [110,111,112]. However, the most striking feature found in children with SARS-CoV-2 is that peripheral blood lymphocyte levels remain in a normal range. This suggests that an active adaptive immune response seems to be promoted, although its magnitude is lower than in adults [113,114]. This could be due to the constitutively higher presence of lymphocytes in children, probably due to the frequent infections contracted in childhood and the high number of lymphoid-based hematopoietic stem and progenitors cells, which promote lymphopoiesis [115]. For this reason, higher levels of T and B cells probably have a considerable suppression capacity in children. Moreover, B lymphocytes producing IL-10 and IgM-type antibodies promote protection through the IgMs, while IL-10 reduce immune-mediated tissue damage [116]. Particularly, it has been shown that memory B Cells developed during previous infections, when properly stimulated, differentiate into plasma cells and rapidly secrete IgMs which can bind and react to different pathogens. Differently, adult MBCs are likely to promote the secretion of antigen-specific antibodies, therefore being less able to adapt to new antigens [106,115,117].

Notwithstanding, all these assumptions given are far from being final or conclusive. A closer look at the characteristics of thjevenile immune system, together with a comprehensive understanding of the viral pathogenic mechanisms acting in such an immature system, could be the key to fully understand COVID-19 pathophysiology in children, and the consequent differential mortality rates. It is only the beginning of our understanding of COVID-19, which appears to not only be a respiratory disorder, but rather a systemic disease involving many organs. Indeed, in the context of SARS-CoV-2 infection and the immune system function, more research is critically needed, and more efforts need to be concerted in order to have a better understanding of, and eventually develop a treatment for SARS-CoV-2.

## Figures and Tables

**Table 1:** List of cytokines, with their respective families, functions and cellular origin.

Cytokine	Family	Type and Function	Cell Sources
IL-1β	IL-1	Pro-inflammatory cytokine; Pyrogenic cytokine; Induction of Macrophages and T cells proliferation and differentiation;	Macrophages, monocytes, fibroblasts
IL-1Ra	IL-1	Anti-inflammatory cytokine; Inhibition of IL-1 activity	Macrophages, monocytes, fibroblasts
IL-2	IL-2	Major growth factor and effector of T cells	T cells
IL-6	IL-6	Pro-inflammatory cytokine; Pyrogenic function;	Macrophages, T cells, endothelial cells
IL-10	IL-10	Anti-inflammatory cytokine; Inhibition of cytokine release and inflammatory response	Monocytes, T cells, B cells
IL-12	IL-12	Pro-inflammatory cytokine; Promotion of the Th1 pathway; Suppression of the Th2 pathway; Activation of NK cells	DCs, Macrophages, Neutrophils
IL-17	IL-17	Promotion of neutrophilic inflammation	Activated T cells, NK cells
IFN-α	IFN-I	Induction of antiviral immunity	pDCs, Monocytes, Leukocytes
IFN-β	IFN-I	Induction of antiviral immunity	pDCs, Monocytes, Fibroblasts
IFN-γ	IFN-II	Pro-inflammatory cytokine; Activation of Macrophages and NK cells	Th1 cells, CTLs, NK cells, activated B cells;
IFN-λ	IFN-III	Induction of antiviral immunity	Activated T cells, NK cells, DCs, Macrophages
TNFα	TNF	Pro-inflammatory cytokine; Pyrogenic cytokine; Induction of the expression of adhesion molecules	Macrophages, T cells, NK cells
IP-10	CXCL10	Interferon-induced chemokine; Induction of recruitment of Macrophages, NK cells and T cells	Monocytes, Endothelial cells

DCs: Dendritic Cells; pDCs: Plamacytoid Dendritic Cells; NK cells: Natural Killer cells; Th1 cells: cell type 1 T cells.
